# Comparative Proteomics Reveals the Spoilage-Related Factors of *Shewanella putrefaciens* Under Refrigerated Condition

**DOI:** 10.3389/fmicb.2021.740482

**Published:** 2021-12-03

**Authors:** Zhengkai Yi, Jing Xie

**Affiliations:** ^1^College of Food Science and Technology, Shanghai Ocean University, Shanghai, China; ^2^Shanghai Professional Technology Service Platform on Cold Chain Equipment Performance and Energy Saving Evaluation, Shanghai Ocean University, Shanghai, China; ^3^National Experimental Teaching Demonstration Center for Food Science and Engineering, Shanghai Ocean University, Shanghai, China; ^4^Shanghai Engineering Research Center of Aquatic Product Processing and Preservation, Shanghai Ocean University, Shanghai, China; ^5^Collaborative Innovation Center of Seafood Deep Processing, Ministry of Education, Dalian, China

**Keywords:** *Shewanella putrefaciens*, spoilage potential, proteomics, intracellular differential expressed proteins, spoilage-related proteins

## Abstract

*Shewanella putrefaciens* is a microorganism with strong spoilage potential for aquatic products. This study aimed to investigate the potential spoilage factors of *S. putrefaciens* by comparative proteomic analysis. The spoilage potential of two strains of *S. putrefaciens* (00A and 00B) isolated from chilled spoiled bigeye tuna was investigated. The results of total volatile basic nitrogen (TVB-N), trimethylamine (TMA) in fish inoculated with *S. putrefaciens*, extracellular protease activity of *S. putrefaciens*, and degradation of fish proteins indicated that the spoilage potential of *S. putrefaciens* 00A was much higher than that of 00B. Fish proteins are usually degraded by spoilage microorganism proteases into small molecular peptides and amino acids, which are subsequently degraded into spoilage metabolites in bacterial cells, leading to deterioration of fish quality. Thus, proteomic analysis of the extracellular and intracellular proteins of 00A vs. 00B was performed. The results indicated that the intracellular differentially expressed protein (IDEP) contained 243 upregulated proteins and 308 downregulated proteins, while 78 upregulated proteins and 4 downregulated proteins were found in the extracellular differentially expressed protein (EDEP). GO annotation revealed that IDEP and EDEP were mainly involved in cellular and metabolic processes. KEGG annotation results showed that the upregulated proteins in IDEP were mainly involved in sulfur metabolism, amino acid metabolism, and aminoacyl-tRNA biosynthesis, while downregulated proteins were related to propanoate metabolism. In contrast, EDEP of KEGG annotation was mainly involved in ribosomes, quorum sensing, and carbohydrate metabolism. Proteins associated with spoilage containing sulfur metabolism (sulfite reductase, sulfate adenylyltransferase, adenylyl-sulfate kinase), amino acid metabolism (biosynthetic arginine decarboxylase, histidine ammonia-lyase), trimethylamine metabolism (trimethylamine-N-oxide reductase), and extracellular proteins (ATP-dependent Clp protease proteolytic subunit) were identified as upregulated. These proteins may play a key role in the spoilage potential of *S. putrefaciens*. These findings would contribute to the identification of key spoilage factors and understanding of the spoilage mechanism of microorganisms.

## Introduction

Bigeye tuna (*Thunnus obesus*) has become one of the most economically valuable fish in the world because of its rich nutritional value and huge production (Wang and Xie, 2019). However, bigeye tuna flesh can easily deteriorate in a short period and lose its nutritional value even under refrigerated conditions (Rossi et al., 2002). Microbial growth and metabolism are vital factors that lead to spoilage including quality loss, the production of off-flavors, and even harmful substances (Ruiz-Capillas and Moral, 2005; Pataca et al., 2021). Although a variety of spoilage bacteria have been found in marine fish, *Shewanella putrefaciens* is considered to be one of the main destructive factors of marine fish under low-temperature storage. *S. putrefaciens* reduces trimethylamine N-oxide (TMAO) to trimethylamine (TMA) during anaerobic respiration (Gao et al., 2019; Qian et al., 2021). In addition, *S. putrefaciens* can also be involved in the metabolism of sulfur-containing amino acids or proteins to produce H_2_S, which is considered to be the main source of fishy odor (Remenant et al., 2015; Wright et al., 2019). These psychrotrophic bacteria should be of concern during the cold chain for the marketing of marine fish such as bigeye tuna.

At low temperatures, extracellular hydrolytic enzymes (including lipases, proteases, and gelatinases) secreted by spoilage bacteria, especially protein hydrolases, are recognized as the main cause of fish spoilage (von Neubeck et al., 2015; Zhu et al., 2017). The protein in fish muscle can be degraded into small-molecule peptides and free amino acids by the microbial extracellular proteolytic enzymes, resulting in deterioration of fish quality (Wang G. et al., 2018). Small peptides and amino acids will be metabolized to ammonia and biogenic amines by the intracellular deaminase and decarboxylase (Zhuang et al., 2021b). Liu et al. (2018a) studied the degradation of sarcoplasmic and myofibrillar protein in cold-storage bighead carp inoculated with different spoilage bacteria, and the results showed that the spoilage ability of strains was consistent with the ability to degrade proteins. A similar study was also performed in chicken (Wang G. et al., 2017). Moreover, the extracellular protease activity of spoilage bacteria has a similar trend to other spoilage indicators (total volatile basic nitrogen, etc.) (Liu et al., 2018b). The spoilage potential of spoilage bacteria is also related to the activity of intracellular proteases, such as amino acid decarboxylase, deaminase, and oxotrimethylamine reductase (Fan et al., 2014; Fu et al., 2018). Therefore, it is of great significance to study the relationship between the extracellular and intracellular proteins and the spoilage potential in spoilage bacteria during cold storage.

The identification of proteins that play an important role in spoilage is essential for understanding the mechanism of bacterial spoilage during cold storage. Comparative proteomic analysis has emerged as a useful quantitative technique to provide relative measurements of bacterial proteins. Based on comparative proteomics, the identification of extracellular secretory proteins of seven strains of toxic and non-toxic *Vibrio parahaemolyticus* reveals the key pathogenic factors of *V. parahaemolyticus*, which is helpful in clarifying its pathogenic mechanism (He et al., 2015). However, there are few studies on the relationship between extracellular/intracellular proteins and spoilage in *S. putrefaciens*.

This study aimed to investigate biochemical parameters (including total volatile basic nitrogen, TMA, and extracellular protease hydrolytic activity) of bigeye tuna fish juice inoculated with *S. putrefaciens* with large differences in spoilage potential during cold storage. Comparative proteomic analysis of *S. putrefaciens* strains of different spoilage potential was used to clarify the key factors related to spoilage. The results of this study are helpful to understand the spoilage mechanism of *S. putrefaciens* in refrigerated seafood and provide a basis for the development of novel preservatives.

## Materials and Methods

### *Shewanella putrefaciens* Strains and Culture Conditions

Bigeye tuna was caught by Zhonglu Ocean Fishing Company Ltd. (Qingdao, Shandong province, China) and stored at −60°C for about 30 days in sterile vacuum polyethylene bags, then transported in perforated polystyrene boxes with dry ice to the laboratory of Shanghai Ocean University. Subsequently, the samples were thawed in vacuum bags for approximately 12 h at 4°C. After thawing, the fish blocks were transferred to aseptic polyethylene bags under aseptic handling and stored aerobically at 4°C until spoilage. Then, 25 grams of spoiled flesh was homogenized with 0.85% sterile saline solution and 10-fold serially diluted. Samples of serial dilutions (0.1 ml) were spread on the surface of plate count agar and incubated at 30°C for 48 h. *S. putrefaciens* 00A and *S. putrefaciens* 00B strains were identified based on 16S rRNA gene sequences and the VITEK^®^ 2 Compact system (bioMérieux, Craponne, France). Strains were stored in tryptone soy broth (TSB) containing 25% glycerine at −80°C. Before use, stains were precultured in brain heart perfusion solution (BHI) for 18 h and then cultured in TSB at 30°C. Bacterial cell cultures grown at the late-logarithmic phase (8 log CFU/ml, OD_600_ ≈ 0.8) were used for inoculation.

### Preparation and Inoculation of Sterile Fish Juice and Fish Block

After thawing, the back muscle of bigeye tuna was homogenized and then mixed with an appropriate amount of distilled water (1,000 ml of water per kg of meat), boiled for 5 min, filtered, and the filter residue removed. Then, 1.6 g/l of TMAO, 40 mg/l of L-cysteine, and L-methionine were added and autoclaved (121°C, 15 min). The final product was sterile bigeye tuna juice. Sterile fish blocks were prepared by soaking in formalin and ethanol solution according to the method of Li Y. et al. (2020). In brief, sterile fish blocks were prepared by soaking in formalin and ethanol solution followed by irradiating with sterile water and draining with UV light.

The sterile fish blocks were soaked in two bacterial suspensions for 10 min and then fished out, to a final concentration of 3–4 log CFU/g. After inoculation, fish blocks were placed in sterile bags for refrigeration at 4°C for 10 days. For fish juice inoculation, sterile fish juice was mixed with two bacterial suspensions separately to obtain an inoculum level of 3–4 log CFU/ml. Non-inoculated sterile fish juice and sterile fish blocks soaked in 0.85% sterile saline solution were used as controls. The fish juice was stored at 4°C for 10 days.

Sterilized fish juice can be in full contact with spoilage bacteria cells and better reflect the spoilage causing ability of spoilage bacteria. The preparation of sterilized fish blocks was used to reflect the ability of spoilage bacteria to degrade fish muscle tissue and protein. The fish juice was used for microbiological growth analysis, TVB-N and TMA analysis, and random sampling and determination every 2 days. The fish blocks were used for flesh myofibrillar protein degradation analysis (sodium dodecyl sulfate-polyacrylamide gel electrophoresis) and muscle microstructure analysis (scanning electron microscope) on days 0, 6, and 10.

### Spoilage Potential Evaluation

#### Microbiological Analysis

Total viable count (TVC) was determined by Liu et al. (2018a). Briefly, after 1 ml of fish juice sample was serially diluted (1:10, sterile saline solution), 0.1 ml was evenly smeared on an iron agar (IA) plate, incubated at 30°C for 48 h, and black colonies were counted. Plate count agar (PCA) was used for the colony count of control samples.

#### Total Volatile Basic Nitrogen and Trimethylamine Analysis

TVB-N was determined according to the method of Wang X. et al. (2018) with an automatic Kjeldahl apparatus (Kjeltec^TM^ 8,400; FOSS Quality Assurance Co., Ltd., Hovedstaden, Denmark). The analysis of TMA was determined according to the Colorimetric Picric Acid Method. Briefly, fish samples and trichloroacetic acid (TCA) were homogenized and mixed. After centrifugation, the supernatant was mixed with formaldehyde, saturated potassium carbonate solution, and toluene. The toluene layer solution and picric acid were mixed thoroughly, and absorbance readings were taken at 410 nm. TVB-N and TMA contents were expressed as mg N/100 ml.

### Protein Degradation

#### Bacterial Extracellular Proteolytic Assay

The activated bacterial culture was inoculated into fresh TSB medium to a final concentration of 3 log CFU/ml and incubated at 4°C. Proteolytic activity was determined using casein as the substrate (Yu et al., 2019). The samples were centrifuged (4,000 g, 20 min, 4°C), and 1 ml of supernatant was added into 1 ml of azocasein substrate solution (2% in 0.02 mol/l phosphate buffer, pH 6.8) to incubate at 40°C for 10 min. The reaction was stopped by 2 ml of 0.4 mol/l TCA solution standing at room temperature for 20 min. Then, the mixture was centrifuged (6,000 g, 8 min, 4°C) to obtain the supernatant. The supernatant (1 ml) was added with Na_2_CO_3_ (5 ml, 0.4 M) and 1 ml of Folin-phenol reagent. The sample was mixed and incubated at 40°C for 20 min and the absorbance at 680 nm with a BioTek Synergy 2 (Winooski, VT, United States).

#### Sodium Dodecyl Sulfate-Polyacrylamide Gel Electrophoresis

The proteolytic activity of *S. putrefaciens* on the tuna flesh myofibrillar proteins was determined using sodium dodecyl sulfate-polyacrylamide gel electrophoresis (SDS-PAGE) analysis. Myofibrillar proteins were extracted according to the method of Liu et al. (2018a). Then, SDS-PAGE was performed according to the method of Qian et al. (2015).

### Scanning Electron Microscopy

Bigeye tuna muscle was cut into the size of 5 mm × 5 mm × 2 mm and was prefixed with 2.5% (v/v) glutaraldehyde at 4°C overnight. Longitudinal sections of freeze-dried bigeye tuna samples were visualized under scanning electron microscopy (SU5000, Hitachi, Tokyo, Japan) with an accelerating voltage of 5 kV.

### Proteome Analysis

#### Isolation of Extracellular and Intracellular Proteins of *Shewanella putrefaciens* Strains and Digestion

For the extraction of intracellular proteins, *S. putrefaciens* strains were cultured in the TSB to late-logarithmic phase (OD_600_ ≈ 0.6), with shaking, at 4°C. Bacterial cultures were centrifuged at 12,000 rpm for 1 min, and the supernatants were used to extract the extracellular proteases, then intracellular proteins were extracted using the Bacterial Protein Extraction Kit (Sangon, Shanghai, China). Extracellular proteases were extracted according to the method previously described with some modifications (Zhu et al., 2020). The supernatants were filtered through a 0.22-μm PES membrane (Millipore, Bedford, MA, United States) to remove the residual bacterial cells. The proteins in the supernatant were precipitated by acetone and TCA.

The dried protein samples were dissolved in redissolved solution (8 M urea/100 mM Tris–HCl). The protein concentrations were measured using a BCA Assay Kit. Subsequently, protein digestion was determined according to Bathke et al. (2019). Briefly, proteins were reduced with 1 mM dithiothreitol (DTT) and alkylated with 40 mM iodoacetamide. Next, proteins were further digested by trypsin (protein to enzyme ratio 100:1) overnight, and the resulting mixture of peptides was desalted and concentrated, and drained.

#### LC-MS/MS and Protein Identification

The samples (12 samples from four groups) were analyzed on a Q Exactive mass spectrometer coupled to an UltiMate 3,000 nano-flow UHPLC. Each sample was performed on a C18 column (3 μm, 100 μm × 20 mm, Thermo Fisher Scientific, Waltham, MA, United States) at a flow rate of 300 nl/min with two solvent systems (buffer A: 3% dimethylsulfoxide (DMSO), 0.1% formic acid and 97% H_2_O; buffer B: 3% DMSO, 0.1% formic acid, and 97% acetonitrile) in 90 min. The full-scan MS spectra ranged from 350 to 1,800 m/z and were acquired with a mass resolution of 70 K, followed by 15 sequential high-energy collisional dissociation (HCD) MS/MS scans with a resolution of 17.5 K. The dynamic exclusion time for repeated ion collection was set at 35 s. HCD collision energy was set to 28, and the filter window for the quad rod is set to 1.6 DA. MS/MS spectral data were retrieved by MaxQuant (V1.6.6) software (Thermo Fisher Scientific, United States) against the *S. putrefaciens* (31682 entries) from UniProtKB.

### Statistical Analysis

All experiments were repeated at least three times. The data were expressed as the mean ± standard deviation. One-way ANOVA was evaluated statistically by using SPSS 19.0 software (Chicago, IL, United States). Functional analysis of differentially expressed proteins according to GO (Gene Ontology), KEGG pathway, and COG (Cluster of Homologous Proteins).

## Results

### Spoilage Potential Evaluation

#### Microbiological Analysis

**FIGURE 1:**
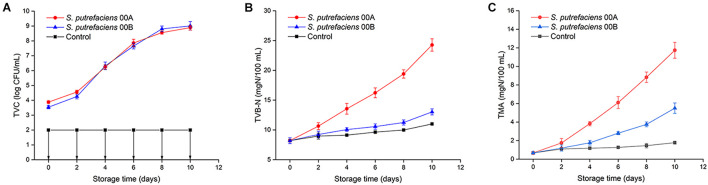
Changes in TVC **(A)**, TVB-N **(B)**, and TMA **(C)** of tuna juice inoculated with *S. putrefaciens* during storage at 4°C.

The initial TVC for fish juice inoculated with *S. putrefaciens* 00A and 00B were 3.88 and 3.54 log CFU/ml, respectively (Figure 1A). Strains 00A and 00B showed similar growth patterns with slow growth in the first 2 days and then reached maximum growth levels of 8.89–9.01 logCFU/ml on day 10 at 4°C. The TVC of fish juice inoculated with two strains showed no significant differences throughout the storage period (*p* > 0.05), while the CK was always below 2 logCFU/ml, indicating a satisfactory preparation of sterile fish juices. Similar growth patterns suggested that both strains had similar cold adaptation in tuna juice.

#### Total Volatile Basic Nitrogen and Trimethylamine

The initial TVB-N content was 8.21 mg/100 g (Figure 1B). The TVB-N values for the CK group did not exceed 11 mg/100 g throughout the storage period. The TVB-N of *S. putrefaciens* 00A increased significantly (*p* < 0.05) throughout the storage period and produced the highest TVB-N, reaching 24.27 mg/100 g on day 10. However, the TVB-N values of the sample inoculated with 00B increased slowly, reaching only 13.03 mg/100 g at the end of storage. Tuna juice inoculated with *S. putrefaciens* 00A produced TVB-N significantly higher than that of 00B (*p* < 0.05), indicating that it had a higher spoilage potential. *Shewanella* species reduced TMAO to TMA, producing a fishy odor in refrigerated marine fish. The changes of TMA in fish juice in CK and inoculation group are shown in Figure 1C. The initial TMA concentration was 0.67 mg/100 g. The TMA value of *S. putrefaciens* 00A- and 00B-inoculated groups significantly increased (*p* < 0.05), reaching 11.74 and 5.50 mg/100 g, while the CK group only reached 1.78 mg/100 g. The TMA of samples inoculated with *S. putrefaciens* 00A was significantly higher than those of the 00B and CK groups from the fourth day of storage (*p* < 0.05).

### Protein Degradation

#### Extracellular Protease Activity

**FIGURE 2:**
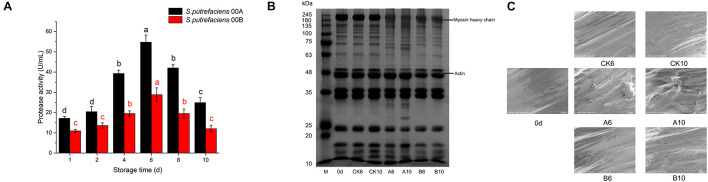
Changes in extracellular protease activity of *S. putrefaciens* **(A)**, SDS-PAGE of myofibrillar protein **(B)**, and SEM of muscle tissue **(C)** during storage at 4°C.

The changes in the extracellular protease activity of the two strains of *S. putrefaciens* 00A and 00B at 4°C are shown in Figure 2A. The extracellular protease activity of *S. putrefaciens* 00A and 00B showed a similar trend, with an increase followed by a decrease. The extracellular protease activity exhibited no significant effect (*p* > 0.05) in the first 2 days and then increased significantly (*p* < 0.05), reaching a maximum value on day 6 in two strains. This was followed by a significant decrease in the last 2 days. In addition, the extracellular protease activity of *S. putrefaciens* 00A was higher than that of 00B throughout the incubation phase and reached the maximum difference on day 8 (115.09% higher in *S. putrefaciens* 00A than in 00B).

#### Sodium Dodecyl Sulfate-Polyacrylamide Gel Electrophoresis

The changes in myofibrillar proteins on days 6 and 10 for the CK and inoculated groups are shown in Figure 2B. There was no significant change in myofibrillar proteins in the CK group after storage for days 6 and 10 compared to samples in day 0. However, the results of SDS-PAGE profiles showed that the intensity of the myosin heavy chain (MHC) of the tuna decreased significantly after being inoculated with *S. putrefaciens* 00A for days 6 and 10. In addition, two bands near 100 kDa became weak, the band near 80 kDa became stronger, and one and three new bands appeared at 63–75 and 25–35 kDa, respectively. This phenomenon was more obvious in the 00A-inoculated samples on day 10 than that on day 6. In contrast, little degradation of myofibrillar proteins in samples inoculated with 00B was observed. In the *S. putrefaciens* 00B groups, no significant degradation of the MHC was detected, while one band was weakened and one was enhanced in the range of 75–100 kDa. Otherwise, no other bands were found to be altered in the 00B groups. SDS results showed that the myofibrillar proteins of bigeye tuna were more severely degraded by *S. putrefaciens* 00A than that of 00B.

#### Scanning Electron Microscope Changes in Muscle Tissue

In this study, the changes in scanning electron microscope (SEM) images of tuna muscle fibers were used to characterize the proteolytic activity of two *S. putrefaciens* strains (Figure 2C). The dynamic changes of myofibril tissues were studied in three groups of samples (CK groups, inoculated with S. putrefaciens 00A groups and 00B groups) on days 0, 6, and 10 under 4°C of storage. On day 0, muscle fibers of the sample showed well-organized, intact, and unbroken morphology. Storage for 6 and 10 days in bigeye tuna samples and cracking of the muscle fiber section occurred to varying degrees. In the CK group stored for 6 and 12 days, the organization of the muscle fibers changed slightly and the structure remained largely undamaged. For samples stored at 6 days in the *S. putrefaciens* 00A group, the organization of the muscle fibers changed, the structure was damaged, and there was bond loss in the myotomes. Bigeye tuna samples inoculated with 00A showed a greater loss of structure of muscle fibers on day 10. In contrast, in tuna samples inoculated with *S. putrefaciens* 00B, muscle fibrous tissue was less disrupted, but a slight disruption of the muscle fibrous structure was also observed on day 10 of inoculation. Similarly, the TEM results confirmed that *S. putrefaciens* 00A was more likely to destroy the muscle fiber structure of tuna than 00B.

### Identification and Bioinformatics Analysis of Intracellular Differential Expressed Protein and Extracellular Differential Expressed Proteins of *Shewanella putrefaciens* 00A and 00B

**FIGURE 3:**
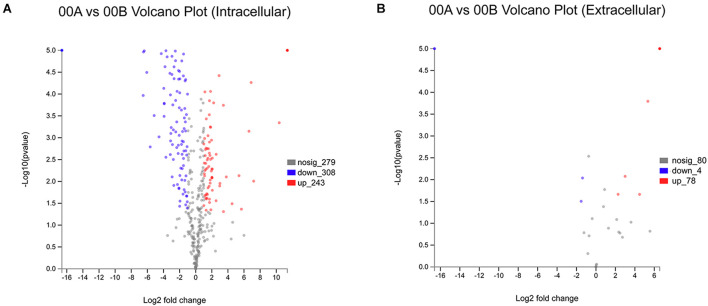
Volcanic map of all identified proteins: volcanic map of intracellular proteins in 00A vs. 00B **(A)**; volcanic map of extracellular proteins in 00A vs. 00B **(B)**. Red points: upregulated proteins (fold change > 2, *p* < 0.05); green points: downregulated proteins (fold change > 0.5, *p* < 0.05); black points: unchanged proteins.

In proteomic analyses, 830 and 162 intracellular and extracellular proteins were identified, respectively, with the *p*-value set at < 0.05 and normalized ratios of > 2 and < 0.5, which allowed 522 and 82 intracellular differential expressed proteins (IDEPs) and extracellular differential expressed proteins (EDEPs) to be classified. As shown in Figure 3A, of the 551 IDEP, 243 were significantly upregulated and 308 were downregulated in the 00A vs. 00B group. In addition, there were 78 upregulated proteins and 4 downregulated proteins in the EDEP (Figure 3B). The IDEP and EDEP are given in Supplementary Tables 1, 2.

**FIGURE 4:**
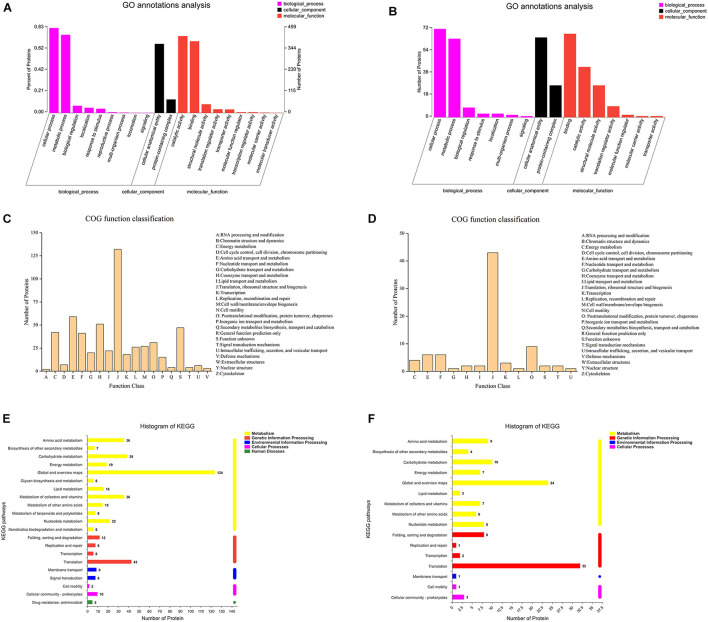
GO annotation analysis of the intracellular **(A)** and extracellular **(B)** differentially expressed proteins in 00A vs. 00B. COG terms of the intracellular **(C)** and extracellular **(D)** differentially expressed proteins in 00A vs. 00B. KEGG pathway analysis of the intracellular **(E)** and extracellular **(F)** differentially expressed proteins in 00A vs. 00B.

As shown in Figures 4A,B, a GO annotation analysis was performed for 522 and 82 differentially expressed proteins in the IDEP and EDEP of the 00A vs. 00B group, respectively. There were three main categories of GO annotation analysis: biological process (BP), cellular component (CC), and molecular function (MF). In IDEP and EDEP, the cellular process and the metabolic process were the two main categories in the BP. The cell anatomy entity was the main item in CC (373 and 65 differentially expressed proteins in IDEP and EDEP of 00A vs. 00B, respectively). In the MF, differential proteins were mainly involved in catalytic activity and binding. In IDEP, GO function revealed that upregulated proteins were related to the oxidation–reduction process, catabolic process, oxidoreductase activity, catalytic activity, and translation regulator activity. Downregulated proteins were related to protein–DNA complex and structural constituent of ribosome (Supplementary Table 3). For BP, differential proteins were upregulated and mainly associated with the primary metabolic process, nitrogen compound metabolic process, and cell periphery (Supplementary Table 4).

Figures 4C,D display the COG function classification. In IDEP, there were a total of 19 categories, of which the top six COG terms were Translation, ribosomal structure and biogenesis, Amino acid transport and metabolism, Coenzyme transport and metabolism, Function unknown, Energy production and conversion, and Nucleotide transport and metabolism. In EDEP, 13 functions were classified, of which the relevant COG terms were Translation, ribosomal structure, and biogenesis.

Pathway category statistical histograms were conducted to show the metabolic pathways of *S. putrefaciens* 00A and 00B (Figures 4E,F). The KEGG pathways of translation, carbohydrate metabolism, amino acid metabolism, and vitamin metabolism were significantly related to IDEP. For EDEP, the main KEGG pathways were translation, carbohydrate metabolism, and amino acid metabolism. In addition, KEGG notes showed that upregulated IDEPs were involved in the pathway of carbon metabolism (sfr01200); sulfur metabolism (sfr00920); arginine and proline metabolism (sfr00330); glycine, serine, and threonine metabolism (sfr00270); and aminoacyl-tRNA biosynthesis (sfr00970) (Supplementary Table 5 and Supplementary Figure 1). KEGG pathways of propanoate metabolism (sfr00640) were the main pathway in IDEP. Furthermore, KEGG analysis indicated that upregulated IDEPs were primarily involved in the ribosome, quorum sensing, biosynthesis of secondary metabolites, carbon metabolism, and microbial metabolism in diverse environments (Supplementary Table 6 and Supplementary Figure 2).

### Spoilage-Related Proteins

As mentioned above, there were differences in the production of TVB-N and TMA and extracellular protease production between *S. putrefaciens* 00A and 00B. The spoilage potential of *Shewanella* spp. was closely related to sulfur metabolism, amino acid metabolism, TMA metabolism, and protease activity. To better understand the reasons for the differences in the spoilage potential of *S. putrefaciens*, the differentially expressed proteins (IDEP and EDEP) associated with spoilage are listed in Table 1. In IDEP, some differentially expressed proteins associated with sulfur metabolism included some upregulated proteins (A4Y7D7, A4Y9Z3, A4Y9Z4, A4Y9 × 2, A4Y9 × 3, A4Y9 × 0, E6XJS8, A4Y5H9, E6XLK8, A0A252F0Q3) and downregulated proteins (A4Y4G8, A4YBG0, A4Y3R7). Several amino acid metabolism-related proteins that may produce biogenic amines, including A4Y5Y9 and A4Y1I3, were upregulated, and no downregulated proteins were identified. Similarly, trimethylamine-N-oxide reductase (O86914, Q8EHI7), a protein associated with TMA metabolism, was upregulated. In addition, proteases containing one upregulated protein (A4Y2S7) and one downregulated protein (A4Y2S8) were listed. In EDEP, the only protease ATP-dependent Clp protease proteolytic subunit (A4Y5I2) was found in the upregulated proteins of 00A vs. 00B.

**TABLE 1:** The spoilage-related proteins in differentially expressed proteins of *S. putrefaciens* 00A vs. 00B.

**Protein name**	**Accession number**	**Location**	**Regulate**
Cysteine desulfurase IscS	A4Y7D7	Intracellular	Up
Biosynthetic arginine decarboxylase	A4Y5Y9	Intracellular	Up
Sulfite reductase	A4Y9Z3	Intracellular	Up
Sulfite reductase [NADPH] flavoprotein alpha-component	A4Y9Z4	Intracellular	Up
Sulfate adenylyltransferase subunit 2	A4Y9X2	Intracellular	Up
Imidazolonepropionase	A4Y1I0	Intracellular	Up
Sulfate adenylyltransferase	A4Y9X3	Intracellular	Up
Adenylyl-sulfate kinase	A4Y9X0	Intracellular	Up
Histidine ammonia-lyase	A4Y1I3	Intracellular	Up
S-Ribosylhomocysteine lyase	A4Y3Y4	Intracellular	Up
ATP-dependent protease ATPase subunit HslU	A4Y2S7	Intracellular	Up
Cysteine desulfurase IscS	E6XJS8	Intracellular	Up
Cysteine–tRNA ligase	A4Y5H9	Intracellular	Up
S-Adenosylmethionine synthase	A4Y3R7	Intracellular	Up
Sulfite reductase [NADPH] hemoprotein beta-component	E6XLK8 A0A252F0Q3	Intracellular	Up
Trimethylamine-N-oxide reductase	O86914	Intracellular	Up
Iron–sulfur cluster insertion protein ErpA	A4Y4G8	Intracellular	Down
Iron–sulfur cluster assembly protein CyaY	A4YBG0	Intracellular	Down
Lipoprotein-releasing system ATP-binding protein LolD	E6XJS2	Intracellular	Down
ATP-dependent protease subunit HslV	A4Y2S8	Intracellular	Down
ATP-dependent Clp protease proteolytic subunit	A4Y5I2	Extracellular	Up
S-Ribosylhomocysteine lyase	E6XLW4	Extracellular	Up
Polyribonucleotide nucleotidyltransferase	A4Y9B5	Extracellular	Up
S-Adenosylmethionine synthase	E6XL87	Extracellular	Up
Fe/S biogenesis protein NfuA	A4YC18	Extracellular	Up

## Discussion

Fish and fish products are highly susceptible to spoilage due to microbial activity. Thus, it is essential to elucidate the mechanisms of spoilage microorganisms in order to detect and control fish product spoilage. *S. putrefaciens* is the specific spoilage organism of many seafood products, such as tuna and Pacific white shrimp, during low-temperature storage (Yang et al., 2020; Lee et al., 2021). In order to characterize the spoilage potential and to reveal the mechanism of two strains of *S. putrefaciens* at refrigerated temperatures, we investigated the spoilage phenotypes of *S. putrefaciens* 00A and 00B under refrigeration and performed proteomics studies on the intracellular and extracellular proteins of the two strains.

In this study, both strains were inoculated into bigeye tuna juice and showed high levels (8.5–9.0 log cfu/ml) at the end of storage. *S. putrefaciens* 00A and 00B grew rapidly and had a similar growth pattern in sterile fish juice, which was consistent with the growth pattern of *S. putrefaciens* inoculated into bighead carp by Liu et al. (2018a). Compared to *S. putrefaciens* 00B, *S. putrefaciens* 00A was an active producer of TVB-N and TMA in chilled storage of bigeye tuna, indicating that 00A has a stronger spoilage potential than 00B. Some researchers also found that individual strains in the same spoilage bacteria species may show different spoilage potentials (Gu et al., 2013; Fu et al., 2018), which revealed that *S. putrefaciens* was considered as a strong spoilage bacterium in some studies (Papadopoulos et al., 2003) but weak spoilage ability in others (Wang H. et al., 2017). TVB-N is mainly composed of ammonia and amines, which were produced by the metabolism of proteins and non-protein nitrogenous compounds by spoilage bacteria (Zhuang et al., 2021a). The difference between the two strains of TVB-N in this study may be caused by the difference in the ability of the strains to metabolize amino acids. TMA, an important indicator of spoilage of aquatic products, is mainly reduced to TMAO by the action of TMA oxide reductase (Zhu et al., 2016). Therefore, *S. putrefaciens* 00A may possess a stronger TMA oxide reductase activity and thus produces more TMA.

Proteases secreted by spoilage bacteria break down proteins into small peptides and free amino acids, which were further broken down in bacterial cells into spoilage metabolites such as nitrogenous compounds and amines (Li Y. et al., 2020; Zhuang et al., 2021a). Therefore, extracellular protease activity was one of the important spoilage indicators of spoilage microbes. In this study, the temporal changes in the extracellular protease activity of *S. putrefaciens* 00A and 00B showed a consistent trend, that is, an increase followed by a decrease and reaching the highest value on day 8. Previous studies showed that the consumption of small-molecule compounds in the early stages leads to bacterial growth and then increase in protease activity (Moradi et al., 2019; Wang and Xie, 2020). In the later stage of the culture, the nutrients in the culture medium were scarce and therefore the protease activity decreased. The protease activity of *S. putrefaciens* 00A was higher than that of 00B, indicating a higher spoilage potential of 00A, which is consistent with the results that 00A produced more TVB-N and TMA. In terms of protein degradation, the hydrolytic activity of *S. putrefaciens* 00A was higher than that of 00B for fish proteins, which was in accordance with the results of extracellular protease activity. The deterioration and softening of fish filets during refrigeration are considered to be the result of muscle protein degradation, associated with various protein hydrolysis systems of microorganisms, which allows spoilage microorganisms to use the internal nutrients of the fish to cause faster spoilage (Ge et al., 2016). Silbande et al. (2016) reported that *S. putrefaciens* readily hydrolyzed myofibrillar proteins of the blunt snout bream. Liu et al. (2018a) determined the protein hydrolytic activity of *S. putrefaciens* in frozen bighead carp and reported that this bacterium was able to degrade myofibrillar proteins, which was consistent with our study. To investigate the damage of the fish muscle myofibril structure by *S. putrefaciens*, SEM changes in muscle tissue were measured in this study. With the increase in storage time, the muscle tissue of fish in the 00A group was more damaged. This may be due to the strong protein hydrolytic activity of the 00A group and the loss of water resulting in the destruction and disorder of the muscle structure (Yu et al., 2020). The destruction of the fish muscle tissue structure also accelerated the process of spoilage bacteria using the fish substrate to cause spoilage. Overall, extracellular protease activity, SDS-PAGE, and SEM showed that *S. putrefaciens* 00A possessed stronger protein hydrolysis activity than 00B.

Proteomics is a potential emerging tool for studying protein expression and has been used to investigate the mechanism of biofilm formation in *S. putrefaciens* under cold stress (Yan and Xie, 2020). In IDEP, upregulation of proteins associated with oxidation–reduction and metabolic processes in 00A vs. 00B indicated that *S. putrefaciens* 00A underwent stronger oxidation–reduction and metabolic activity than 00B. The upregulated intracellular expression proteins of 00B were mostly used in the structural composition of ribosomes. In addition, IDEP is involved in amino acid and nucleotide catabolism, allowing bacteria to use amino acids and nucleotides as a source of energy. The production of energy promotes translation in bacterial cells, which activates the formation of ribosomal structures (Gupta et al., 2013). KEGG annotation results indicated that the upregulated proteins in IDEP (00A vs. 00B) were mainly involved in carbon metabolism, sulfur metabolism, amino acid metabolism (glycine, serine, threonine, cysteine, methionine, and arginine), and aminoacyl-tRNA biosynthesis (Supplementary Table 5 and Supplementary Figure 1). The enrichment of the sulfur metabolism pathway indicated that bacteria may produce more sulfur-containing metabolites, accelerating spoilage of aquatic products and producing malodor (Fu et al., 2018), which also predicts that 00A may produce more H_2_S. Proteins involved in the amino acid metabolic pathway were upregulated, indicating that the bacteria may produce more spoilage metabolites such as ammonia and amines (Zhuang et al., 2021b), which also provide energy for bacterial growth (Valgepea et al., 2017). The protein associated with aminoacyl-tRNA biosynthesis was upregulated in 00A vs. 00B, which may be related to the synthesis of protein, contributing to the formation of stable biofilm and spoilage metabolic activities in bacteria (Yan and Xie, 2020). In EDEP, 78 upregulated proteins but only 4 downregulated proteins were in the 00A group, compared with the 00B group. In GO classification, nitrogen metabolism was one of the main items involved in upregulated proteins. The metabolism of nitrogen-containing compounds by bacteria may lead to the accumulation of ammonia and amines, which also explained the higher production of TVB-N by *S. putrefaciens* 00A metabolism in fish juice than 00B. As shown in Supplementary Table 6 and Supplementary Figure 2, the ribosomal pathway was the most enriched, with many ribosomal proteins found in EDEP. Three proteins related to quorum sensing (QS) were upregulated. QS was shown in many studies to regulate the formation of bacterial biofilm and induce bacterial spoilage potential (Zhao et al., 2016; Fu et al., 2018). The enrichment of the carbohydrate metabolic pathway suggested that polysaccharides may be converted to glucose and fructose *via* transferases or transport systems, which can be further metabolized by the glycolytic pathway (Zhong et al., 2019). In conclusion, *S. putrefaciens* 00A secreted more extracellular proteins than 00B, which may indicate that 00A can be able to use the aquatic substrate more quickly to accelerate spoilage.

Bacterial spoilage activity in aquatic products is usually associated with protein degradation, sulfur metabolism, amino acid metabolism, and TMA metabolism (Remenant et al., 2015). To better understand the spoilage potential of *S. putrefaciens* 00A and 00B, Table 1 shows the list of spoilage-related differentially expressed proteins. Upregulated proteins related to sulfur metabolism may be important in causing sulfur-containing spoilage metabolites (Leustek et al., 2000). Sulfate is converted to adenosine 5′-phosphosulfate (APS) through ATP consumption by sulfate adenylyltransferase. APS is then converted to 3′-phosphonoadenosine-5′-phosphate sulfate (PAPS) by the action of adenylyl-sulfate kinase, which continues to be reduced to sulfite by phosphonoadenosine phosphate reductase. Finally, sulfite is catalyzed by sulfite reductase to form sulfide (Li J. et al., 2020). In this study, multiple enzymes related to the sulfur metabolic pathway were found to be upregulated (A4Y9Z3, A4Y9 × 3, A4Y9 × 0), which means that 00A could produce more sulfide (Cordente et al., 2009; Konopka et al., 2015). In addition, we also identified amino acid metabolism-related proteins including arginine decarboxylase (A4Y5Y9) and histidine deaminase (A4Y1I3) among the upregulated proteins, which may be related to the production of ammonia and putrescine (Jorgensen et al., 2000; Zhuang et al., 2021b). Since the aquatic products are rich in TMAO, it is reduced to TMA by TMA oxide reductase produced by the spoilage bacteria, producing a fishy odor (Dos Santos et al., 1998; Wang et al., 2019). Trimethylamine-N-oxide reductase (O86914) was found in the upregulated proteins of 00A vs. 00B, which at least partially explained the fact that fish juice inoculated with *S. putrefaciens* 00A produced more TMA than that inoculated with 00B. Furthermore, an extracellular protease, the ATP-dependent Clp protease hydrolytic subunit (A4Y5I2), was identified in EDEP, whose main activity was the hydrolysis of proteins into peptides and amino acids in the presence of ATP and magnesium ions (Porankiewicz et al., 1999). In addition, it plays an important role in the degradation of misfolded proteins (Bewley et al., 2009; Wang et al., 2010). The upregulation of ATP-dependent Clp protease hydrolytic subunit in group 00A may be the main reason for its strong extracellular protease activity. Overall, most of the differentially expressed proteins associated with spoilage in 00A vs. 00B were upregulated. Although these identified proteins were reported to be associated with bacterial spoilage in the literature, further experimental validation was needed in future studies (such as related genes deletion, suppression, and overexpression).

## Conclusion

In summary, this study explored the spoilage phenotype and comparative proteomics analyses of intracellular and extracellular proteins between *S. putrefaciens* 00A and 00B. The results indicated that *S. putrefaciens* 00A exhibited a stronger spoilage potential (in terms of TVB-N, TMA, extracellular protease activity, fish protein degradation, and tissue structure destruction) than 00B. Proteomic analysis revealed that differentially expressed proteins were mainly involved in amino acid metabolism, sulfur metabolism, and carbohydrate metabolism. The differentially expressed proteins related to spoilage were almost all upregulated in 00A vs. 00B, mainly including sulfite reductase, sulfate adenylyltransferase, adenylyl-sulfate kinase, arginine decarboxylase, histidine ammonia-lyase, trimethylamine-N-oxide reductase, and ATP-dependent Clp protease proteolytic subunit, which may be associated with bacterial spoilage potential. This research would help to understand the spoilage mechanism of *S. putrefaciens*.

## Data Availability Statement

The original contributions presented in the study are included in the article/Supplementary Material, further inquiries can be directed to the corresponding author/s.

## Author Contributions

ZY: conceptualization, methodology, software, investigation, and writing. JX: validation, formal analysis, writing—review and editing, examination, and funding acquisition. Both authors contributed to the article and approved the submitted version.

## Conflict of Interest

The authors declare that the research was conducted in the absence of any commercial or financial relationships that could be construed as a potential conflict of interest.

## Publisher’s Note

All claims expressed in this article are solely those of the authors and do not necessarily represent those of their affiliated organizations, or those of the publisher, the editors and the reviewers. Any product that may be evaluated in this article, or claim that may be made by its manufacturer, is not guaranteed or endorsed by the publisher.
