# Supplementary material for: Comparative Proteomics Reveals the Spoilage-Related Factors of Shewanella putrefaciens Under Refrigerated Condition
Source: Front Microbiol. 2021 Dec 3;12:740482. doi: 10.3389/fmicb.2021.740482 (PMC8678035; doi:10.3389/fmicb.2021.740482)
Supplement: Supplementary file 1 [file Data_Sheet_1.docx]

(A)


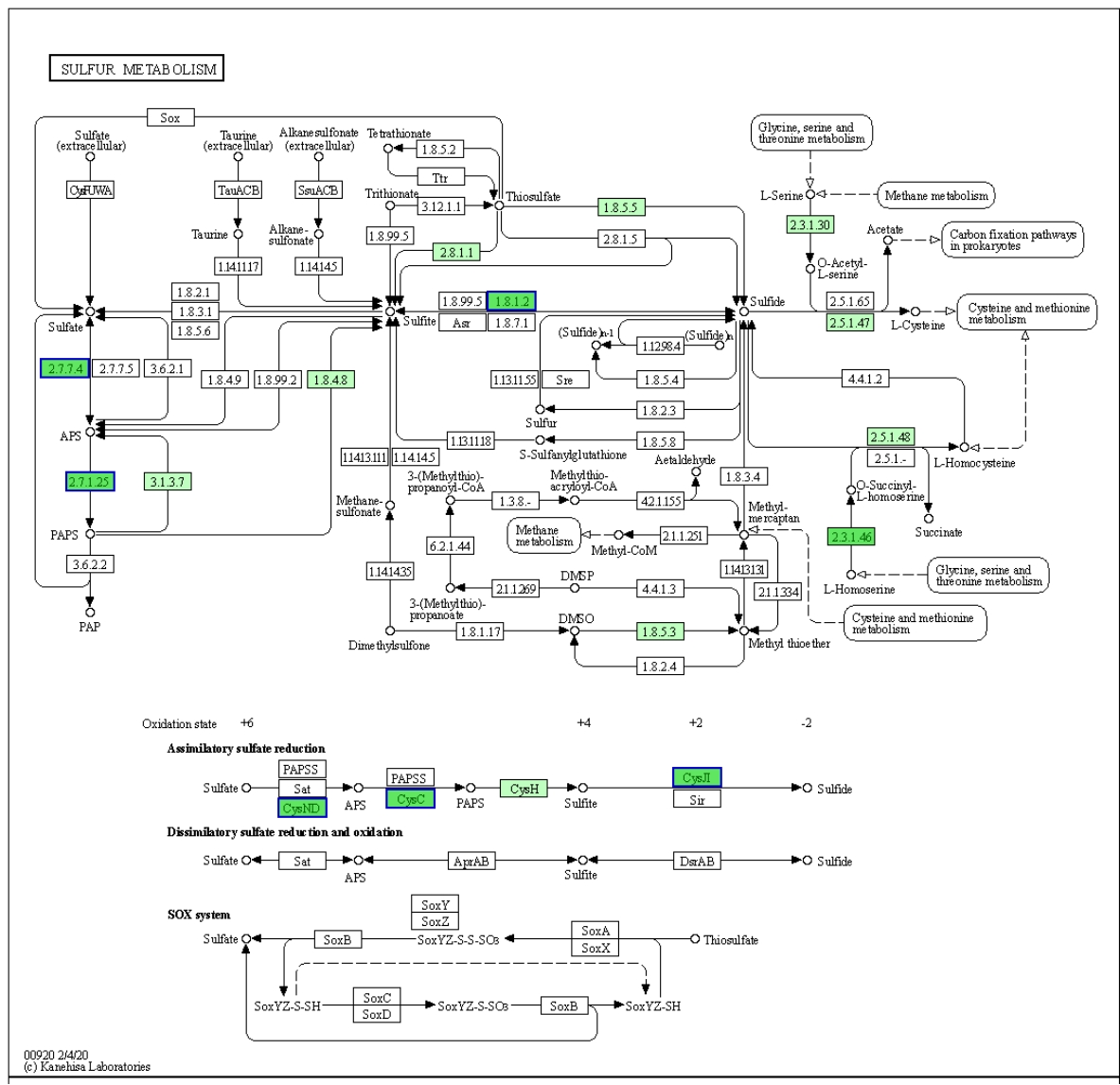


(B)


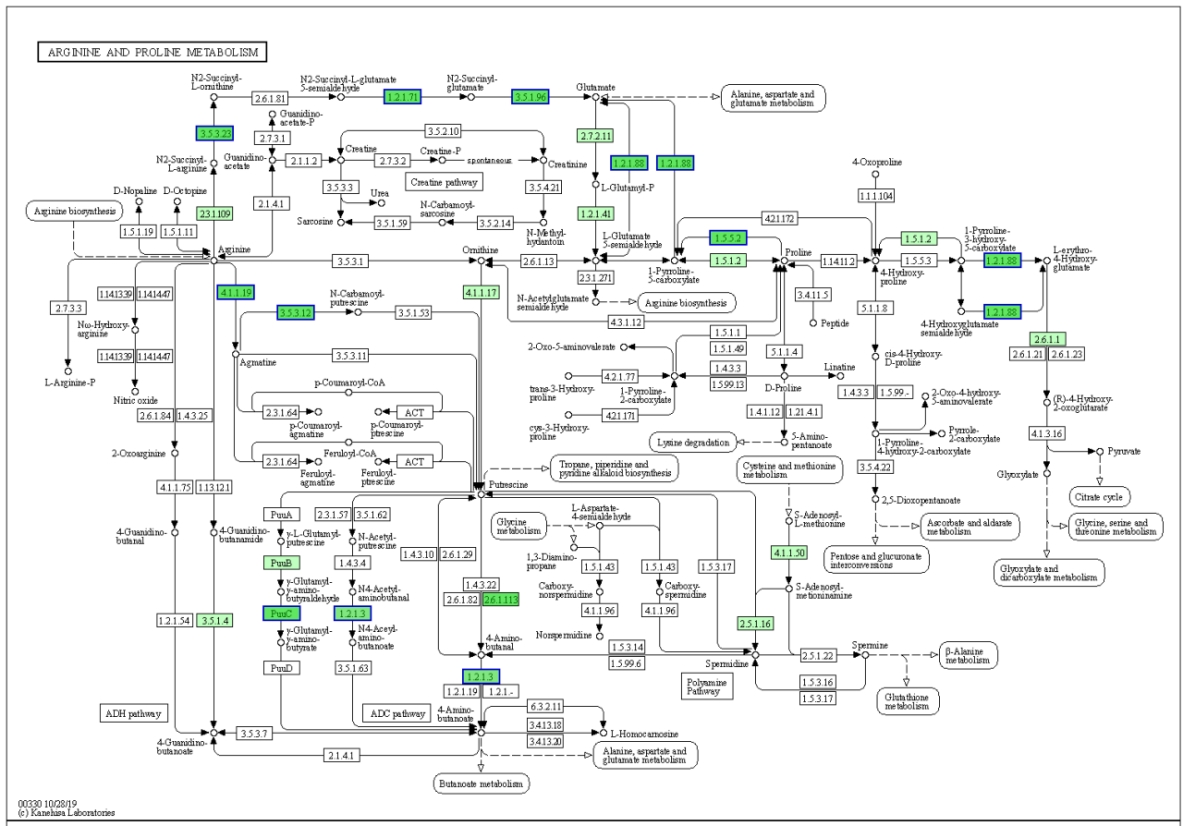


(C)


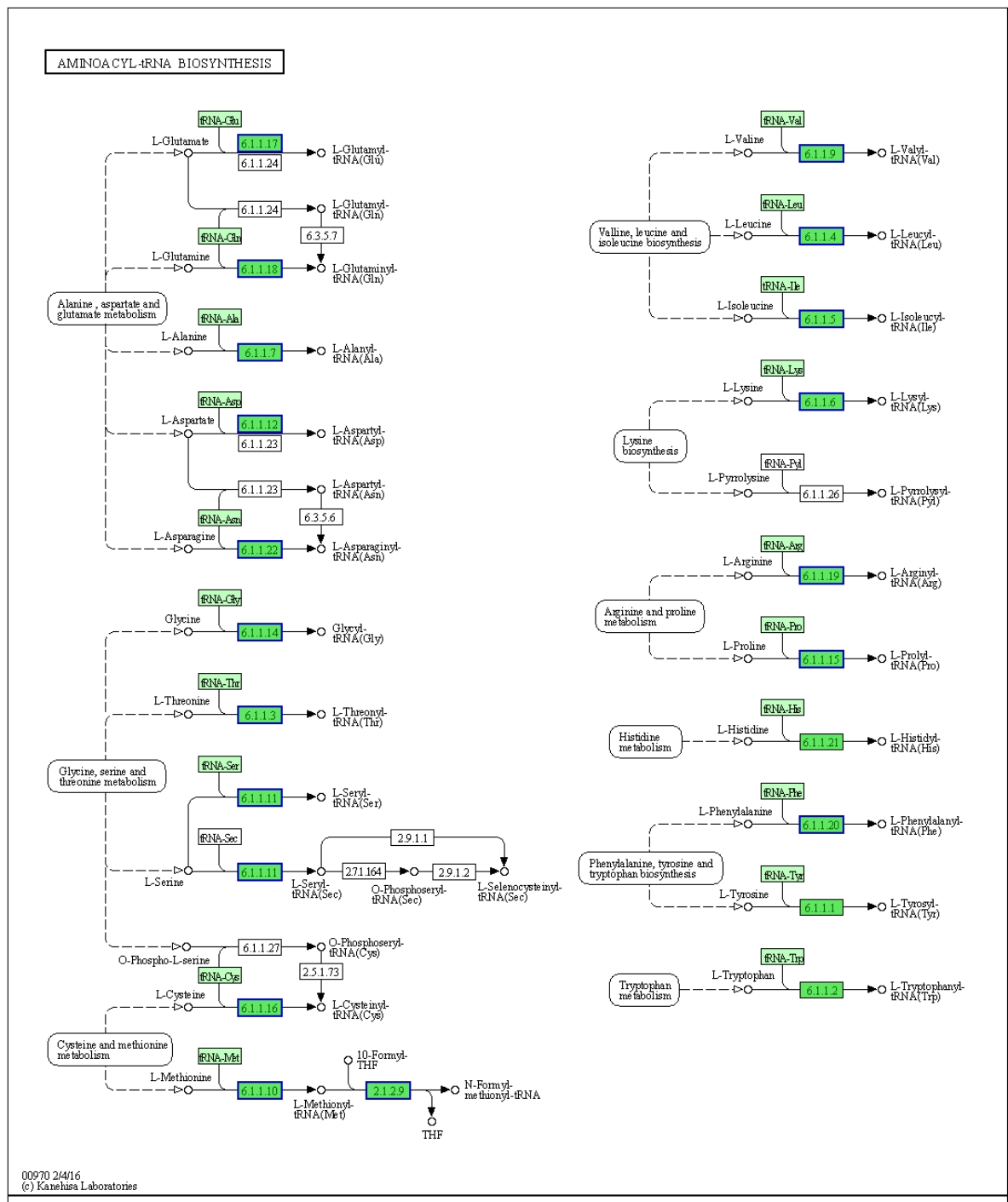


**Supplementary Figure 1.** KEGG pathway of sulfur metabolism (A); arginine and proline metabolism (B); and aminoacyl-tRNA biosynthesis (C) in intracellular differentially expressed proteins. Deep green: protein enriched to the pathway; light green: species enriched to the pathway; red ring or blue ring: protein set.

(A)


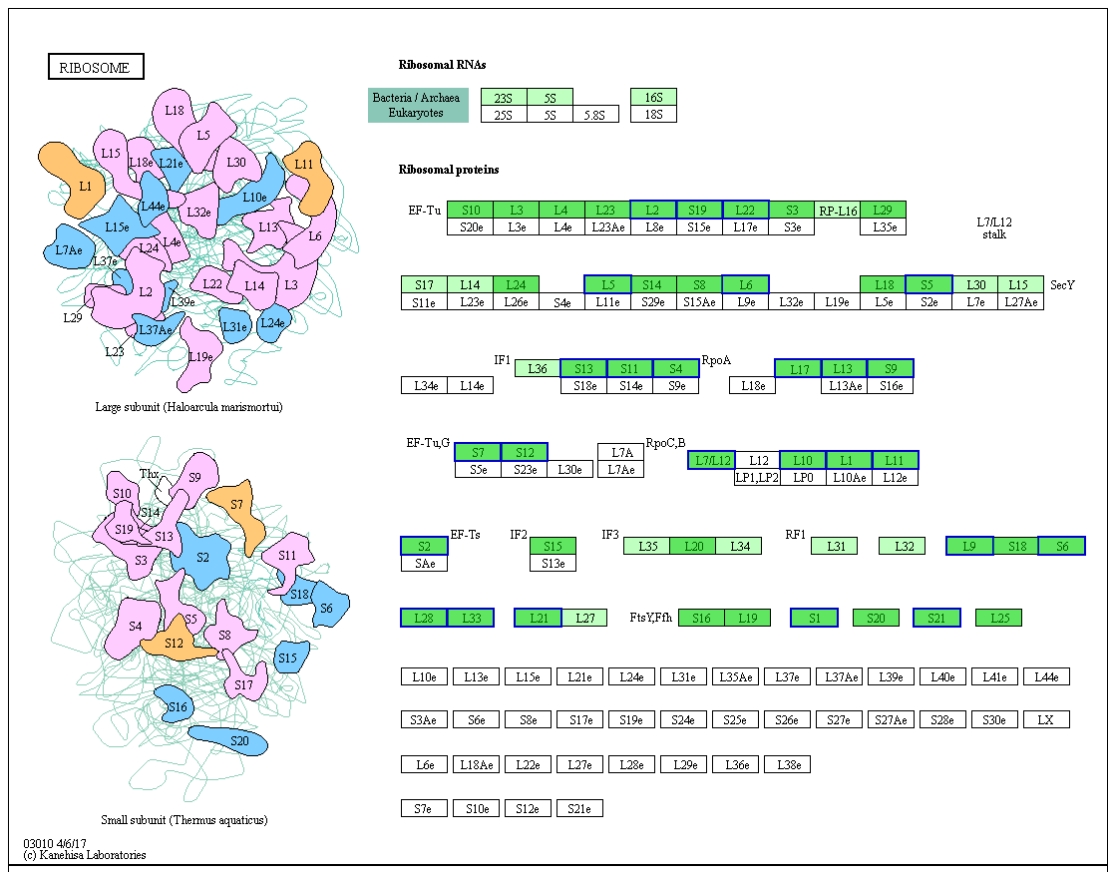


(B)


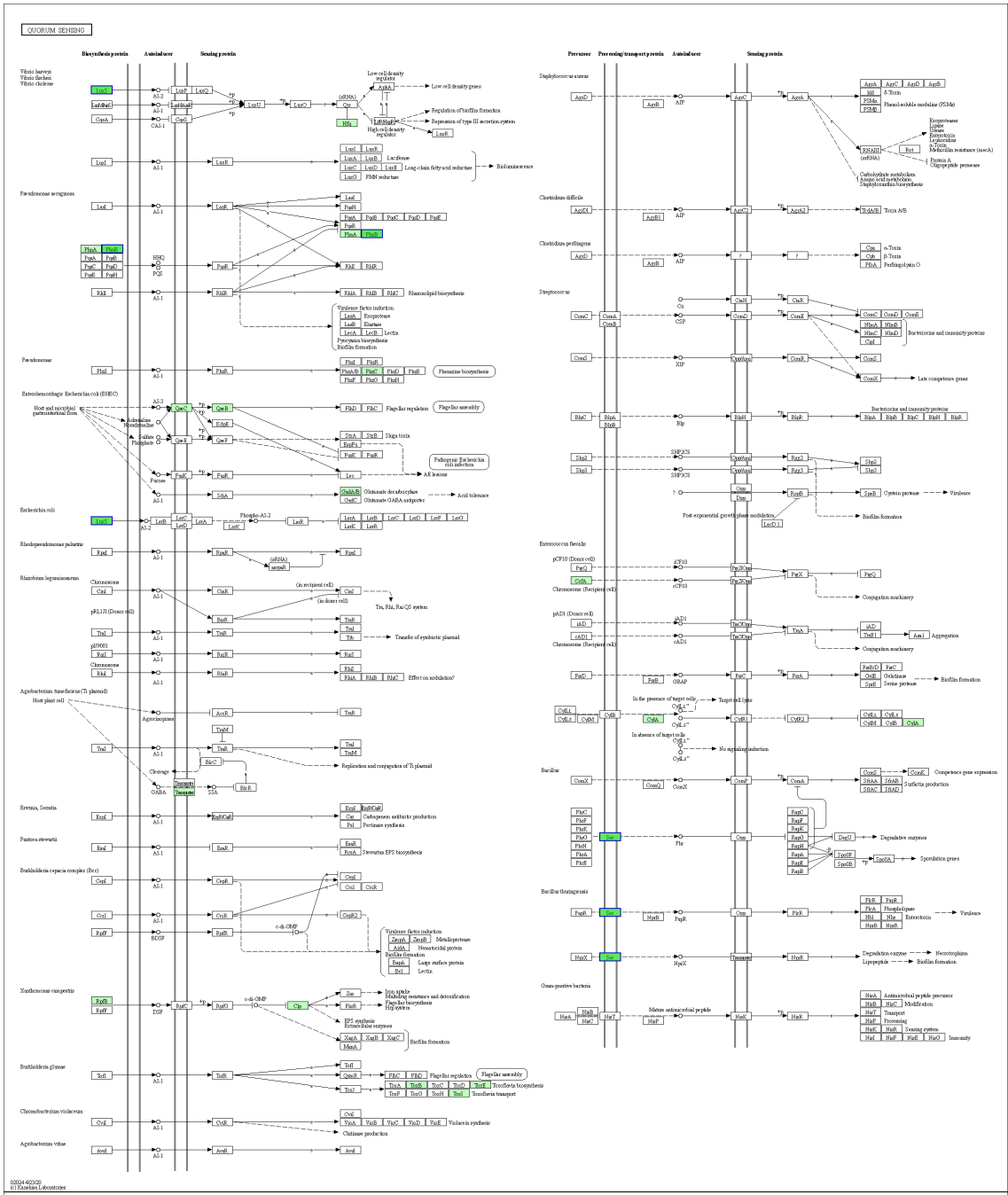


(C)


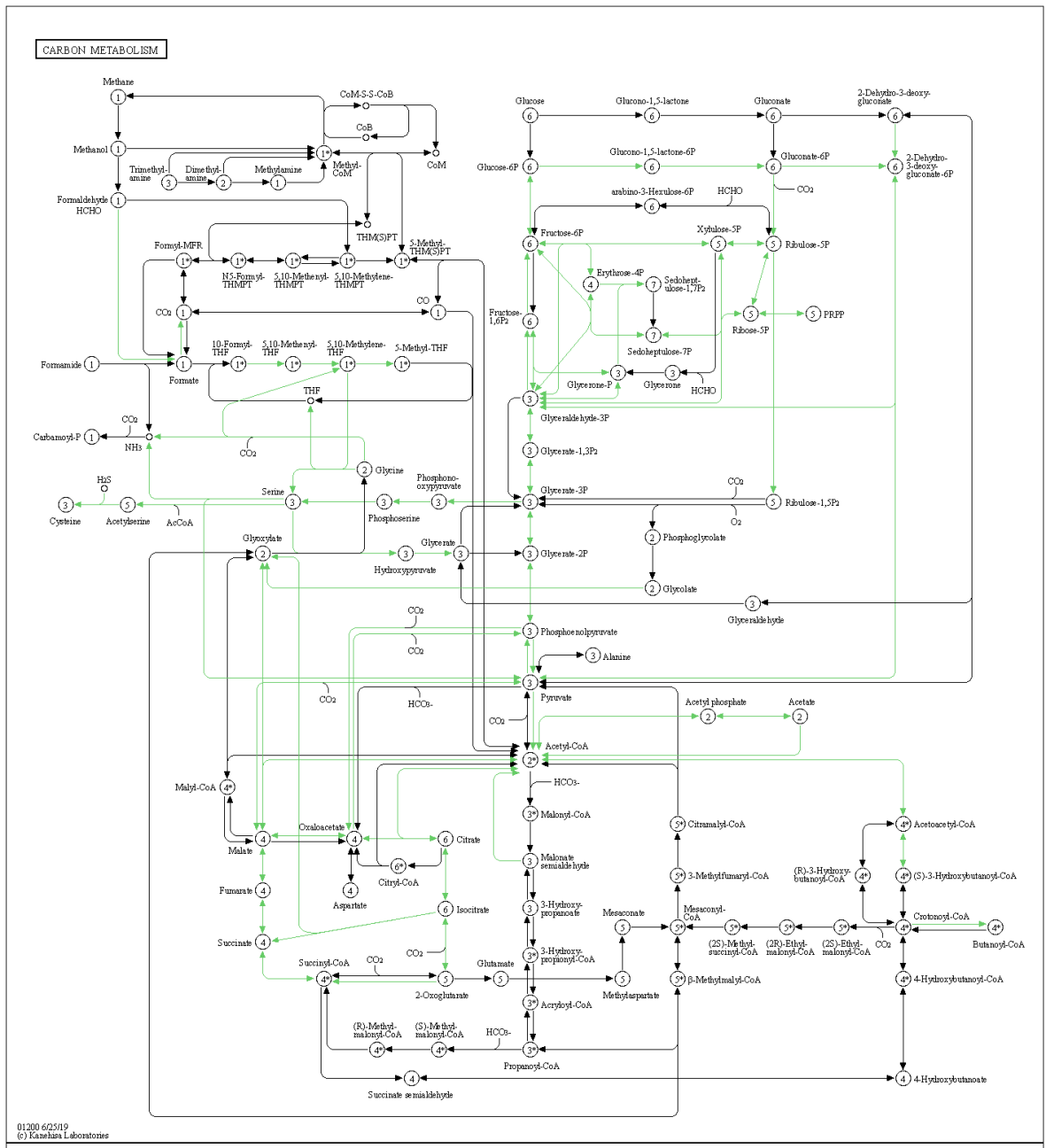


**Supplementary Figure 2.** KEGG pathway of ribosome (A); quorum sensing (B); and Carbon metabolism (C) in extracellular differentially expressed proteins. Deep green: protein enriched to the pathway; light green: species enriched to the pathway; red ring or blue ring: protein set.
