# Supplementary material for: Comparative Proteomics Reveals the Spoilage-Related Factors of Shewanella putrefaciens Under Refrigerated Condition
Source: Front Microbiol. 2021 Dec 3;12:740482. doi: 10.3389/fmicb.2021.740482 (PMC8678035; doi:10.3389/fmicb.2021.740482)
Supplement: Supplementary file 4 [file Table_3.docx]

**Supplementary Table 3.** GO function of intracellular differentially expressed proteins

| **GO ID** | **GO Description** | **Protein Numbers** | **Up Numbers** | **Down Numbers** |
| --- | --- | --- | --- | --- |
| GO:0042440 | pigment metabolic process | 11 | 6 | 5 |
| GO:0006950 | response to stress | 65 | 36 | 29 |
| GO:0032993 | protein-DNA complex | 7 | 1 | 6 |
| GO:0016853 | isomerase activity | 26 | 12 | 14 |
| GO:0009337 | sulfite reductase complex (NADPH) | 5 | 4 | 1 |
| GO:0140110 | transcription regulator activity | 16 | 11 | 5 |
| GO:0097159 | organic cyclic compound binding | 143 | 65 | 78 |
| GO:1901363 | heterocyclic compound binding | 143 | 69 | 74 |
| GO:0055114 | oxidation-reduction process | 54 | 40 | 14 |
| GO:0016829 | lyase activity | 35 | 10 | 25 |
| GO:0009628 | response to abiotic stimulus | 42 | 20 | 22 |
| GO:0009056 | catabolic process | 50 | 32 | 18 |
| GO:1990904 | ribonucleoprotein complex | 45 | 15 | 30 |
| GO:0003735 | structural constituent of ribosome | 44 | 15 | 29 |
| GO:0051716 | cellular response to stimulus | 40 | 17 | 23 |
| GO:0042221 | response to chemical | 25 | 9 | 16 |
| GO:0016491 | oxidoreductase activity | 41 | 30 | 11 |
| GO:0016874 | ligase activity | 58 | 48 | 10 |
| GO:0140096 | catalytic activity, acting on a protein | 19 | 17 | 2 |
| GO:0090079 | translation regulator activity, nucleic acid binding | 21 | 18 | 3 |
