# Supplementary material for: Comparative Proteomics Reveals the Spoilage-Related Factors of Shewanella putrefaciens Under Refrigerated Condition
Source: Front Microbiol. 2021 Dec 3;12:740482. doi: 10.3389/fmicb.2021.740482 (PMC8678035; doi:10.3389/fmicb.2021.740482)
Supplement: Supplementary file 5 [file Table_4.docx]

**Supplementary Table 4**. GO function of extracellular differentially expressed proteins

| **GO ID** | **GO Description** | **Protein Numbers** | **Up Number** | **Down Number** |
| --- | --- | --- | --- | --- |
| GO:0071944 | cell periphery | 34 | 32 | 2 |
| GO:0016020 | membrane | 31 | 29 | 2 |
| GO:0003735 | structural constituent of ribosome | 27 | 27 | 0 |
| GO:1990904 | ribonucleoprotein complex | 27 | 27 | 0 |
| GO:0036094 | small molecule binding | 16 | 14 | 2 |
| GO:0005576 | extracellular region | 27 | 25 | 2 |
| GO:0006950 | response to stress | 18 | 17 | 1 |
| GO:0009986 | cell surface | 12 | 12 | 0 |
| GO:0097367 | carbohydrate derivative binding | 11 | 10 | 1 |
| GO:0005515 | protein binding | 23 | 22 | 1 |
| GO:0090079 | translation regulator activity, nucleic acid binding | 9 | 9 | 0 |
| GO:0043167 | ion binding | 22 | 21 | 1 |
| GO:0044238 | primary metabolic process | 73 | 72 | 1 |
| GO:0009058 | biosynthetic process | 61 | 60 | 1 |
| GO:0050789 | regulation of biological process | 16 | 15 | 1 |
| GO:0006807 | nitrogen compound metabolic process | 69 | 68 | 1 |
| GO:0009056 | catabolic process | 11 | 11 | 0 |
| GO:0016787 | hydrolase activity | 12 | 11 | 1 |
| GO:0046034 | ATP metabolic process | 3 | 3 | 0 |
| GO:0140098 | catalytic activity, acting on RNA | 11 | 10 | 1 |
