# Supplementary material for: Comparative Proteomics Reveals the Spoilage-Related Factors of Shewanella putrefaciens Under Refrigerated Condition
Source: Front Microbiol. 2021 Dec 3;12:740482. doi: 10.3389/fmicb.2021.740482 (PMC8678035; doi:10.3389/fmicb.2021.740482)
Supplement: Supplementary file 6 [file Table_5.docx]

**Supplementary Table 5**. KEGG annotation analysis of intracellular differentially expressed proteins

| **Pathway ID** | **Description** | **Protein numbers** | **Up numbers** | **Down numbers** |
| --- | --- | --- | --- | --- |
| map00640 | Propanoate metabolism | 21 | 5 | 16 |
| map00630 | Glyoxylate and dicarboxylate metabolism | 24 | 12 | 12 |
| map01212 | Fatty acid metabolism | 16 | 8 | 8 |
| map01200 | Carbon metabolism | 57 | 39 | 18 |
| map00920 | Sulfur metabolism | 17 | 15 | 2 |
| map00230 | Purine metabolism | 38 | 17 | 21 |
| map00330 | Arginine and proline metabolism | 9 | 6 | 3 |
| map00260 | Glycine, serine and threonine metabolism | 18 | 12 | 6 |
| map00270 | Cysteine and methionine metabolism | 12 | 9 | 3 |
| map00970 | Aminoacyl-tRNA biosynthesis | 28 | 20 | 8 |
