# Supplementary material for: Comparative Proteomics Reveals the Spoilage-Related Factors of Shewanella putrefaciens Under Refrigerated Condition
Source: Front Microbiol. 2021 Dec 3;12:740482. doi: 10.3389/fmicb.2021.740482 (PMC8678035; doi:10.3389/fmicb.2021.740482)
Supplement: Supplementary file 7 [file Table_6.docx]

**Supplementary Table 6.** KEGG annotation analysis of extracellular differentially expressed proteins

| **Pathway ID** | **Description** | **Protein numbers** | **Up numbers** | **Down numbers** |
| --- | --- | --- | --- | --- |
| map03010 | Ribosome | 27 | 27 | 0 |
| map03018 | RNA degradation | 6 | 5 | 1 |
| map00920 | Sulfur metabolism | 4 | 4 | 0 |
| map02024 | Quorum sensing | 3 | 3 | 0 |
| map00230 | Purine metabolism | 8 | 8 | 0 |
| map01110 | Biosynthesis of secondary metabolites | 18 | 17 | 1 |
| map00260 | Glycine, serine and threonine metabolism | 4 | 4 | 0 |
| map01230 | Biosynthesis of amino acids | 6 | 6 | 0 |
| map01200 | Carbon metabolism | 8 | 7 | 1 |
| map01120 | Microbial metabolism in diverse environments | 11 | 10 | 1 |
